# β-blockers and metabolic modulation: unraveling the complex interplay with glucose metabolism, inflammation and oxidative stress

**DOI:** 10.3389/fphar.2024.1489657

**Published:** 2024-12-20

**Authors:** Szymon Drygała, Michał Radzikowski, Mateusz Maciejczyk

**Affiliations:** ^1^ Department of Hygiene, Epidemiology and Ergonomics, Medical University of Bialystok, Bialystok, Poland; ^2^ Biochemistry of Civilisation Diseases’ Students’ Scientific Club at the Department of Hygiene, Epidemiology and Ergonomics, Medical University of Bialystok, Bialystok, Poland

**Keywords:** anti-inflammatory effects, antioxidant properties, beta-adrenoreceptor antagonists, β-blockers, carvedilol, metabolic diseases, glucose control, nebivolol

## Abstract

The growing burden of metabolic disorders manifested by hypertension, type 2 diabetes mellitus, hyperlipidemia, obesity and non-alcoholic fatty liver disease presents a significant global health challenge by contributing to cardiovascular diseases and high mortality rates. Β-blockers are among the most widely used drugs in the treatment of hypertension and acute cardiovascular events. In addition to blocking the receptor sites for catecholamines, third-generation β-blockers with associated vasodilating properties, such as carvedilol and nebivolol, provide a broad spectrum of metabolic effects, including anti-inflammatory and antioxidant properties and a favorable impact on glucose and lipid metabolism. This review aims to report the impact of β-blockers on metabolic modulation based on available literature data. We present an overview of β-blockers and their pleiotropic properties, discuss mechanisms by which these drugs affect cellular metabolism and outline the future perspectives. The influence of β-blockers on glucose metabolism, insulin sensitivity, inflammation and oxidative stress is complex and varies depending on the specific β-blocker used, patient population and underlying health conditions. Recent evidence particularly highlights the potential role of vasodilatory and nitric oxide-mediated properties of nebivolol and carvedilol in improving glycemic control, insulin sensitivity, and lipid metabolism and mitigating oxidative stress and inflammation. It suggests that these drugs may be potential therapeutic options for patients with metabolic disorders, extending beyond their primary role in cardiovascular management.

## 1 Introduction

Metabolic diseases encompass a range of conditions that involve disruptions in normal metabolic processes within the body. These diseases can be congenital or acquired and are becoming increasingly prevalent globally. Diabetes, characterized by high blood sugar levels, is a well-known metabolic disorder that affects millions worldwide. The World Health Organization (WHO) estimates that over 400 million adults are expected to suffer from diabetes by 2030 (Shaw et al., 2010; Veiseh et al., 2015). Obesity, which results from an imbalance between energy intake and expenditure leading to excess adipose tissue, is closely linked to diabetes and other metabolic conditions. Atherosclerosis, a condition where lipoproteins accumulate in arterial walls, is another metabolic disorder contributing to cardiovascular diseases (CVDs) (Shi et al., 2019). CVDs include conditions such as coronary heart disease, cerebrovascular disease, peripheral arterial disease, myocardial infarction, stroke and heart failure (Olvera Lopez et al., 2023). CVDs are the leading cause of death worldwide (Vaduganathan et al., 2022) and a significant complication of metabolic disorders, with a complex connection between systemic inflammation, immune dysregulation, metabolic abnormalities and cardiovascular risk factors leading to progressive cardiovascular damage. Metabolic syndrome, characterized by a collection of metabolic disorders such as abdominal adiposity, dyslipidemia, hypertension and increased fasting blood glucose, is closely associated with the development of CVDs (Shi et al., 2023). Pharmacological treatment options for cardiovascular diseases comprise many groups of medicines. Diuretics, calcium channel blockers, renin-angiotensin-aldosterone system (RAAS) inhibitors, statins and anticoagulants, as well as modern antidiabetic cardiovascular drugs such as sodium-glucose transport protein 2 (SGLT2) inhibitors, dipeptidyl peptidase 4 (DPP-4) inhibitors and glucagon-like peptide-1 receptor agonists (GLP-1) are used in prevention of CVDs and acute cardiovascular events treatment (Bazargani et al., 2018; Wojtasińska et al., 2023). Β-blockers are one of the drugs used to prevent CVDs and manage acute events. Such medicines are widely used in the treatment of hypertension, a risk factor for CVDs, as well as for stable ischemic heart disease, acute coronary syndrome, heart failure and atrial fibrillation.

Β-blockers are medicines with well-established positions in clinical practice. They have been used mainly in cardiology for over 40 years. This group includes many molecules with diverse pharmacokinetic and pharmacodynamic properties. That is why researchers worldwide constantly investigate the potential use of β-blockers in different therapeutic areas and indications. The mechanism of action of β-blockers involves their competitive antagonism of beta-adrenergic receptors, leading to a reduction in the effects of catecholamines such as epinephrine and norepinephrine. This action decreases the heart rate, myocardial contractility and systemic vascular resistance, thereby reducing cardiac workload and oxygen demand. Additionally, β-blockers have been shown to have antiarrhythmic properties by modulating the heart’s electrical conduction system (Weber et al., 1974). These drugs have several additional effects, including a broad spectrum of metabolic effects such as anti-inflammatory properties, oxidative stress attenuation, or a positive impact on glucose and lipid metabolism. Those pleiotropic properties suggest that the positive outcome of β-blockers in conditions such as CVDs could result from a more complex mechanism than a β-receptor blockade.

This review aims to present the potential impact of β-blockers on metabolic modulation based on available literature data.

## 2 Overview of β-blockers and their classification

The mechanism of action of β-blockers involves their competitive antagonism of adrenergic stimulation of beta-adrenoceptors through structural similarities to catecholamines, which stimulate beta-adrenoceptors (β1, β2 and β3) as well as alpha-adrenoceptors (α1 and α2). While β3-adrenoreceptors remain inactive under basal conditions, they can induce negative inotropic effects during intense adrenergic stimulation and mediate processes like lipolysis and thermogenesis. The cardiovascular effects of catecholamines involve interactions with the central nervous system, sympathetic ganglia, heart, peripheral arteries and kidneys. In the sympathetic nervous system, central α2-adrenoreceptor activation inhibits sympathetic activity, modulated by postganglionic neurons where noradrenergic release is regulated by presynaptic α2-adrenoreceptor stimulation and β2-adrenoreceptor activation. The heart predominantly contains β1-adrenoreceptors, which, along with β2, trigger positive inotropic, chronotropic, lusitropic and dromotropic effects via cAMP-dependent pathways. Adrenoceptors on arterial smooth muscle cells mediate vasoconstriction (α1) and vasodilation (β2). At the renal level, renin release from juxtaglomerular cells is mediated by β1, while sympathetic stimulation of the adrenal medulla leads to epinephrine release. Beyond the cardiovascular system, adrenoceptor-dependent mechanisms regulate carbohydrate metabolism, insulin release, lipolysis, skeletal muscle contraction and bronchodilation (Gorre and Vandekerckhove, 2010).

The chemical structure of β-blockers is of organic compounds consisting of an aryloxypropanolamine side chain to which an aromatic or heteroaromatic ring is attached (Bekhradnia and Ebrahimzadeh, 2012). β-blockers are classified based on their selectivity for β-adrenergic receptors, vasodilatory potential and other pharmacological properties. The classifications of β-blockers include nonselective, beta-1 selective and those with additional vasodilatory effects (Northfield and Manallack, 2007). Nonselective β-blockers, such as propranolol, block both beta-1 and beta-2 adrenergic receptors, while beta-1 selective β-blockers, i.e., metoprolol, primarily target beta-1 receptors. β-blockers with additional vasodilatory effects, such as carvedilol and nebivolol, exhibit vasodilatory properties in addition to their beta receptors blocking effects (Northfield and Manallack, 2007; Diaconu et al., 2019). These classifications are important for understanding the pharmacological properties and clinical applications of different β-blockers, providing insights into their selectivity, vasodilatory potential and chemical characteristics.

Β-blockers treatments have led to concerns about their effectiveness in patients with comorbidities and any adverse metabolic effects. It should be noted that adverse metabolic effects have been observed with “traditional” β-blockers such as atenolol and metoprolol. The next-generation of β-blockers, i.e., carvedilol and nebivolol, are changing the way β-blockers are perceived in clinical practice. These agents have been shown to have beneficial metabolic effects and efficacy in cardiovascular disease management (Agabiti Rosei and Rizzoni, 2007; Marketou et al., 2017). Absorption, distribution, metabolism and elimination vary between nonselective and selective β-blockers. The pharmacodynamic and pharmacokinetic differences between nonselective and selective β-blockers have clinical implications for their use in various conditions, including heart failure, hypertension and arrhythmias. The distinct hemodynamic effects and receptor selectivity of these agents may influence their efficacy, safety and tolerability in different patient populations. Additionally, the pharmacokinetic properties of β-blockers can impact their potential for drug interactions and adverse effects, highlighting the importance of individualized treatment approaches (Huck et al., 2022).

## 3 Pleiotropic properties of β-blockers

**FIGURE 1:**
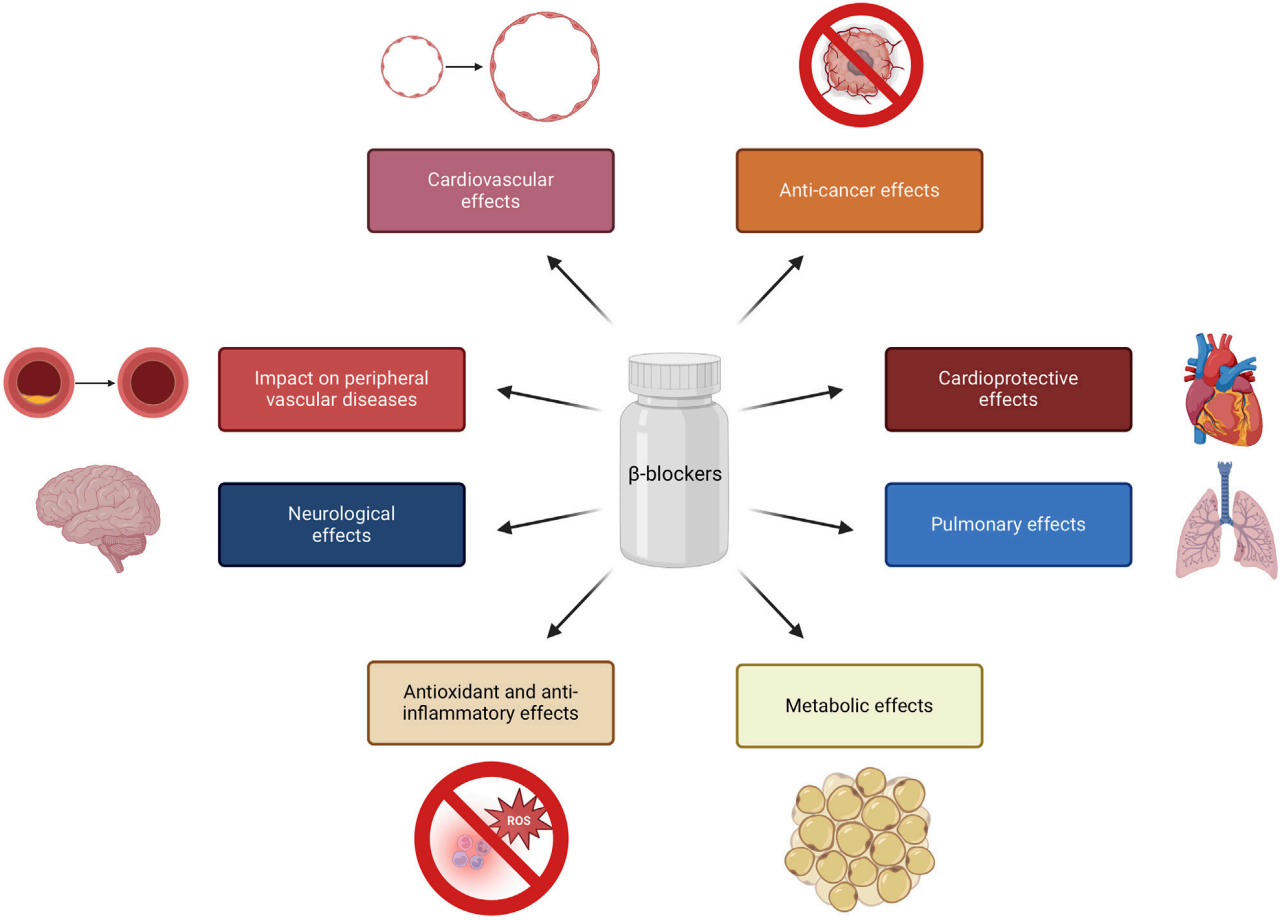
Pleiotropic properties of β-blockers. Created using biorender.com.

Β-blockers, traditionally known for their role in CVDs management, have been found to exhibit pleiotropic properties that extend beyond their primary pharmacological effects. These additional properties have implications in different physiological systems and clinical conditions, such as antioxidant and anti-inflammatory effects, vasodilatory properties, neuroprotective effects and potential impacts on pulmonary function. Furthermore, β-blockers have been associated with potential effects on peripheral vascular diseases, neuroleptic-induced acute akathisia and hepatocellular carcinoma outcomes (Figure 1) (Lima et al., 2004; Sasso and Rockey, 2021; Chang et al., 2019).

Below are selected examples of the pleiotropic effects of β-blockers:

- Metabolic effects: β-blockers have been linked to metabolic effects, such as weight gain and potential implications in diabetes mellitus (Hossain et al., 2018; Tomiyama and Yamashina, 2014), while several studies have suggested that vasodilatory β-blockers may have beneficial effects on glucose and lipid metabolism (Deedwania, 2011; Peixoto et al., 2020; Fongemie and Felix-Getzik, 2015). β-blockers have been associated with potential antioxidant and anti-inflammatory effects, which may contribute to their protective role in cardiovascular health (Ladage et al., 2013; Fatani et al., 2015; Haas et al., 2016).
- Cardiovascular effects: certain β-blockers, such as nebivolol, have vasodilatory properties, contributing to their potential to reduce systemic vascular resistance and improve endothelial function (Huck et al., 2022; Fumagalli et al., 2020; Pedersen and Cockcroft, 2007). This group of drugs have been also recognized for their cardioprotective effects, such as reducing myocardial oxygen consumption and inhibiting renin secretion (Bain, 2018; Haas et al., 2016; Imbaby et al., 2014).
- Neurological effects: some studies have suggested that nonselective β-blockers may have neuroprotective properties, potentially impacting neurological outcomes (Kadoi and Saito, 2010; Amirshahrokhi and Niapour, 2022). β-blockers presented efficacy in migraine management by normalizing cortical network variability (CNV), reducing the dependence of evoked cortical potentials on the intensity of auditory stimuli, showing a high affinity for 5-hydroxytryptamine (5-HT) receptors 2B and 2C and inhibiting nitric oxide production by blocking inducible nitric oxide synthase (iNOS) (Siniatchkin et al., 2007; Sandor et al., 2000; Pradhan et al., 2018; Ramadan, 2004).
- Pulmonary effects: cardioselective β-blockers have been shown to have a range of effects on pulmonary function, such as lowering the risk of bronchoconstriction with no impact on exacerbation rate (Duffy et al., 2017; van Gestel et al., 2009; Preveden et al., 2021; Devereux et al., 2024). Mitogen-activated protein kinases (MAPK) inactivation by β-blockers leads to decreased expression of MUC5AC, a mucin associated with mucosal hypersecretion, particularly in the context of cigarette smoke exposure (Zhou et al., 2014).
- Anti-cancer effects: The activation of β-adrenergic receptors can lead to the development and progression of cancer metastases. Specifically, it has been reported that stimulation of tumor β-adrenergic receptors increases the production of several factors that promote metastasis, including vascular endothelial growth factor (VEGF), matrix metalloproteinases (MMP-2 and MMP-9), and pro-inflammatory cytokines such as IL-6 and IL-8 (Cole et al., 2015; Makale et al., 2017). Therefore, β-blockers are hypothesized to alter the tumor microenvironment, thereby providing a protective role in cancer (Wrobel et al., 2020; Na et al., 2018). β-blockers reduce the activity of important signaling pathways (mitogen-activated protein kinase (MAPK), cyclic guanosine monophosphate/protein kinase G (cGMP/PKG), epidermal growth factor receptor (EGFR), cyclooxygenase-2 (COX-2), VEGF), and transcription factors (nuclear factor kappa-light-chain-enhancer of activated B cells (NFkB) and cAMP response element-binding protein (CREB), signal transducer and activator of transcription 3 (STAT3)) which are both important in carcinogenesis (Carnet Le Provost et al., 2023).

These pleiotropic properties of β-blockers highlight their multifaceted impact on a range of physiological systems and clinical conditions, extending beyond their primary role in cardiovascular management.

## 4 Metabolic effects of selected β-blockers

β-blockers exert their effects through specific mechanisms involving the interaction between the drug’s structure and biological activity. When considering how β-blockers influence cellular metabolism, it is essential to delve into the intricate details of their pharmacological actions. It is well known that activation of β-adrenergic receptors promotes cell proliferation, cardiomyocyte hypertrophy and apoptosis (Tanner et al., 2020; He et al., 2017; Zhang et al., 2017). Thus, blockade of β-receptors could mitigate adverse effects related to β-adrenergic activation. β-blockers have been shown to induce apoptosis in various cancer cell lines, which may be beneficial in the context of tumor growth inhibition (Zhou et al., 2016). Similarly, the anti-apoptotic properties of β-blockers have been confirmed in different cardiac conditions, suggesting that while this group of drugs can promote apoptosis in cancer cells. They can also protect cardiomyocytes from apoptotic signals during ischemic events (Ibáñez et al., 2011). In the context of hypertrophy and cardiac remodeling, β-blockers have been shown to affect signaling pathways that regulate cell growth and survival (Hashemi et al., 2015).

**FIGURE 2:**
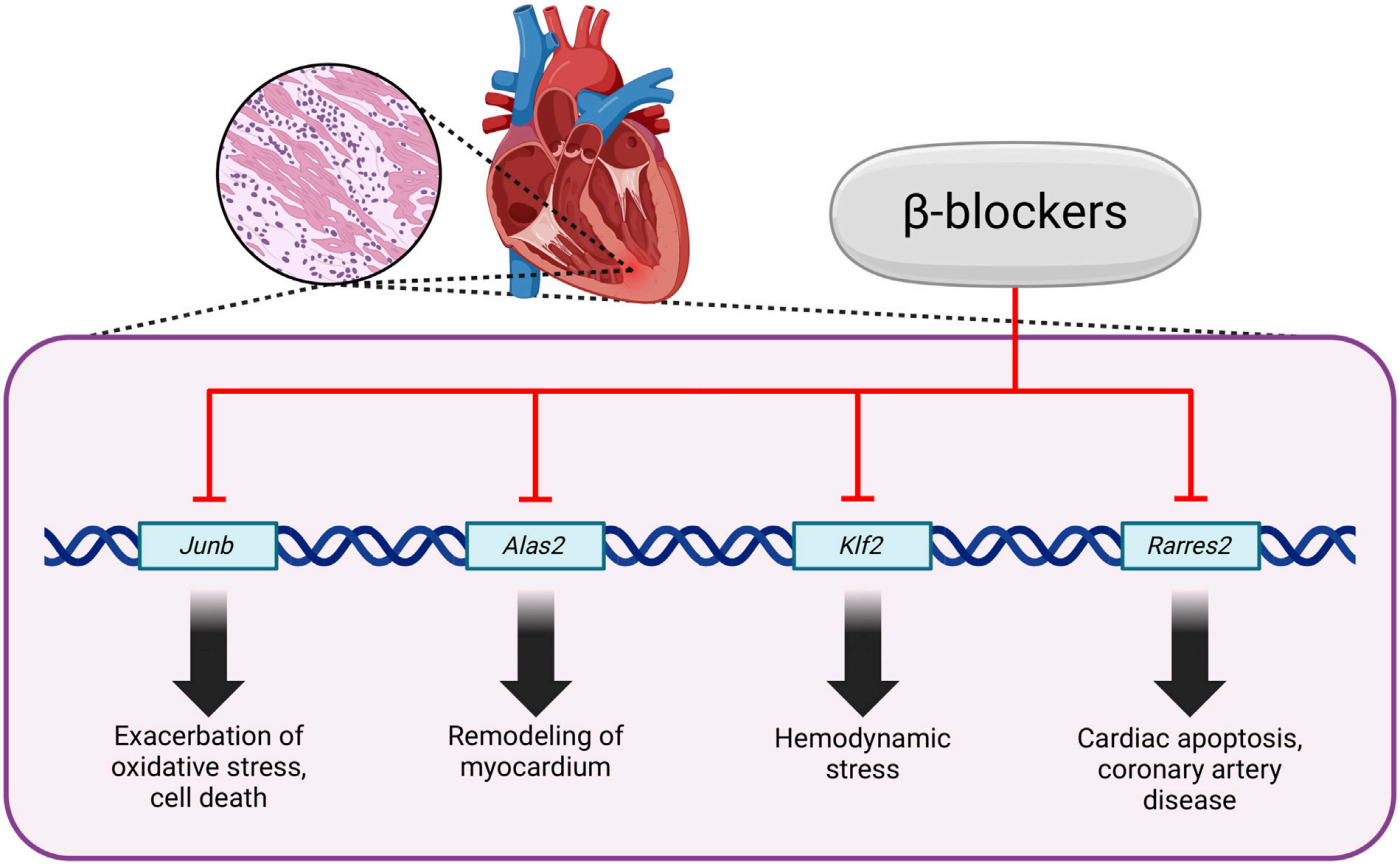
Regulation of gene expression by β-blockers. Created using biorender.com.

One significant aspect to explore is how these medications impact cellular processes at the molecular level, especially concerning metabolic pathways and signaling cascades. Research has shown that β-blockers can affect cellular metabolism by modulating gene expression related to metastasis, inflammation, cell proliferation and angiogenesis. These medications can influence the activation of specific genes, thereby altering the metabolic activity of cells. Experimental research has pinpointed specific genes, such as *Alas2*, *Junb*, *Klf2* and *Rarres2*, the expressions of which can be repressed by β-blockers. These genes involve oxidative stress, hypertrophy, cardiac remodeling, vascular remodeling and apoptosis. For example, *Alas2* may exacerbate oxidative stress and cell death. *Junb*, a transcriptional regulator, is associated with cardiac remodeling, while *Klf2* responds to hemodynamic stress. *Rarres2* has been linked to cardiac apoptosis and coronary artery disease (Figure 2). Additionally, β-blockers exert their effects primarily through the blockade of β-adrenergic receptors, leading to a cascade of intracellular signaling events that ultimately influence gene expression mediated by transcription factors like MEF2 (myocyte enhancer factor 2), which is a crucial regulator of genes involved in cardiac hypertrophy and myocyte survival. It has been concluded that the transcriptomic changes induced by β-blockers are closely linked to alterations in MEF2 expression, suggesting that the responsiveness of MEF2 to β-blockade is context-dependent and may vary with treatment duration and intensity (Hashemi et al., 2015; Tobin et al., 2017). A better understanding of how these genes are regulated by β-adrenergic and MEF2 pathways could provide insights into the mechanisms of heart failure and potential therapeutic targets (Tobin et al., 2017; Peixoto et al., 2020; Sawicki et al., 2015; Lee et al., 2006).

Additionally, β-blockers have been found to impact local muscular metabolic properties, potentially impairing endurance exercise capacity, especially with acute treatment (Ladage et al., 2013). Nebivolol for instance has a unique hemodynamic profile characterized by reduced systemic vascular resistance and improved left ventricular function. This alteration in vascular resistance and cardiac function can have downstream effects on cellular metabolism, influencing nutrient delivery and utilization within cells (Prisant, 2008). In the context of β-blockers and their effects on metabolism, it is crucial to consider the broader implications in conditions such as obesity and hypertension. While β-blockers are effective antihypertensive agents, concerns have been raised regarding their potential adverse effects on metabolic parameters such as lipid profiles and insulin sensitivity, as well as their propensity to induce weight gain in some individuals (Pischon and Sharma, 2001). These metabolic effects can significantly affect overall cellular function and energy regulation, highlighting the intricate interplay between β-blockers and cellular metabolism. Furthermore, the use of β-blockers in managing conditions such as hypertension and heart failure has been associated with improved cardiovascular outcomes and exercise capacity. β-blockers have been shown to reduce mortality through various mechanisms, including the blockade of adrenergic receptors and reductions in heart rate, which can profoundly affect cellular metabolism and energy utilization (Cullington et al., 2012). Additionally, the selective targeting of beta receptors by certain β-blockers can lead to favorable metabolic profiles and beneficial effects on endothelial function and renal protection, further emphasizing the intricate relationship between these medications and cellular metabolism (Tomiyama and Yamashina, 2014). In the realm of cancer research, investigations into the potential role of β-blockers in glioma treatment have shed light on the complex interplay between these medications and cellular processes. While preclinical studies have provided limited evidence for the use of β-blockers in glioma therapy, they have identified potential pathways for targeting glioma cells, suggesting a possible link between β-blockers and cellular proliferation and survival mechanisms (Tewarie et al., 2020).

## 5 Glucose metabolism and insulin sensitivity

Several mechanisms related to adrenergic stimulation may influence insulin sensitivity and glucose metabolism. In healthy individuals, insulin facilitates vasodilation and enhances blood flow to skeletal muscles, which correlates with increased glucose uptake (Steinberg and Baron, 2002). However, insulin-mediated vasodilation is impaired in individuals with insulin resistance, leading to reduced glucose uptake in peripheral tissues. Additionally, acute sympathetic nervous system activation can decrease insulin-stimulated glucose uptake through vasoconstriction, primarily mediated by α1-adrenergic and α2-pathways (Li et al., 2021; Straub and Sharp, 2012; Lembo et al., 1994; Scherrer and Sartori, 1997; Tappy et al., 1995). This occurs through the activation of potassium channels, which results in membrane hyperpolarization of pancreatic β-cells. Specifically, the opening of ATP-sensitive potassium channels reduces the intracellular calcium concentration by inhibiting voltage-dependent calcium channels, thereby preventing the exocytosis of insulin granules (Li et al., 2021; Zabuliene and Ilias, 2024; Straub and Sharp, 2012). β-blockers, particularly nonselective ones, can diminish the first phase of insulin secretion from pancreatic β cells, potentially due to the inhibition of β2-mediated insulin release (Lithell et al., 1992; Sarafidis and Bakris, 2006). Furthermore, sympathetic activation promotes gluconeogenesis and glycogenolysis while inhibiting glycogen synthesis in the liver (Nonogaki and Iguchi, 1997). As α-adrenergic receptors play a significant role in humans, unopposed α activity during β-blockade could enhance hepatic glucose output, thereby elevating the risk of developing type 2 diabetes (Sarafidis and Bakris, 2006; Lund-Johansen et al., 1992).

The impact of β-blockers on glucose metabolism has been a subject of interest in many studies (Table 1). Certain β-blockers, particularly nonselective β-blockers, may be associated with adverse effects on glucose metabolism and insulin sensitivity, potentially leading to an increased risk of developing or exacerbating diabetes (Tomiyama and Yamashina, 2014). Conversely, other research has suggested that certain β-blockers, such as nebivolol or carvedilol, may have more favorable effects on glucose metabolism and insulin sensitivity. The GEMINI study showed that carvedilol affects glucose metabolism more favorably than metoprolol. The study suggested that patients with diabetes mellitus could benefit from treatment with carvedilol due to decreased insulin resistance (Bakris et al., 2004; Kveiborg et al., 2006). These results support the notion that vasodilating β-blockers have a neutral impact on glycemic control and improve insulin sensitivity when compared to agents that solely block β receptors. The neutral effects of contemporary β-blockers on glycemic regulation and insulin sensitivity may be attributed to their α-blocking or β2-stimulating actions, which promote vasodilation and consequently enhance blood flow to skeletal muscle. Therefore, the favorable impact of carvedilol on glycemic parameters may also be partially explained by its hemodynamic effects (Tomlinson et al., 1988; Fongemie and Felix-Getzik, 2015; Ozyildiz et al., 2017; Manrique et al., 2011). Moreover, potassium (K+) channels expressed in pancreatic β-cells and peripheral insulin-sensitive tissues, which play a key role in glucose metabolism, are intensely modulated by carvedilol, establishing a link between K+ channels and the drug’s effects on glucose control. The effects of carvedilol on various K+ channels, including Kv, KAch, KATP and K2P, may have a positive effect on glucose homeostasis, contributing to clinical efficacy in the treatment of patients with hypertension and type 2 diabetes (Li, 2022).

**TABLE 1:** Summary of selected studies describing the metabolic effect of β-blockers.

Study	Drug compound/Setting	Study type	Results	Conclusions
Rizos et al. (2003)	Atenolol 50 mg/d or nebivolol 5 mg/d. After 12 weeks, pravastatin (40 mg/d) was added in hypertensive patients with dyslipidemia	Clinical trial	• Atenolol decreased triglyceride levels by 19% and lipoprotein(a) by 30%, • Atenolol and nebivolol decreased serum high-sensitivity CRP levels by 14% and 15%, respectively, • Nebivolol reduced the HOMA index by 20%. The addition of pravastatin to all patients decreased: • total cholesterol by 21%, • low-density lipoprotein cholesterol by 28%, • apolipoprotein-B by 22%, • apolipoprotein-E by 15%; • homocysteine levels and by 17% • CRP by 43%.	Nebivolol is appropriate therapy in hypertensive patients with hyperlipidemia and carbohydrate intolerance.
Metwally et al. (2020)	Nebivolol 2.5–10 mg/d or carvedilol 3.125–25 mg/d for 12 weeks in patients with non-diabetic and non-ischemic cardiomyopathy with heart failure	Clinical trial	Nebivolol reduces: • fasting glucose, • levels of fasting insulin • HOMA-IR.	Nebivolol improves insulin resistance-related variables.
Ozyildiz et al. (2017)	Carvedilol 25 mg/d or nebivolol 5 mg/d for 4 months in patients with essential hypertension	Clinical trial	Carvedilol and nebivolol decreased: • serum glucose, • insulin, • HOMA-IR, • HDL, • LDL, • total cholesterol, • apolipoprotein.	Carvedilol and nebivolol have similar favorable effects on glucose metabolism, insulin resistance and lipid profile.
Zepeda et al. (2012)	Carvedilol 12.5 mg/d or nebivolol 5 mg/d for 12 weeks in patients with essential hypertension	Clinical trial	• Patients treated with carvedilol show 28.1% and 23.6% lower levels of 8-isoprostane and erythrocyte MDA, • FRAP and GSH/GSSH ratios show 31.5% and 29.6% higher levels in the carvedilol group, • NO_2_ concentration was 62.1% higher in nebivolol group.	Nebivolol increases nitric oxide bioavailability, while carvedilol reinforces the antioxidant system.
Lin et al. (2020)	normal saline, saline + propranolol (2 mg/kg), saline + insulin (2 U/kg), or saline + glucose (2 g/kg) was intraperitoneally delivered to ischemia or sham rats	Animal study	Propranolol alleviated the postischemic changes, including: • MDA and caspase 3 activity, • CRP and COX-2 astrocyte-associated GFAP, • macrophage/microglia lineage-associated CD68 and IRF8, • hyperglycemia and hyperinsulinemia, • downregulation of Microtubule-Associated Protein 2 and tight junction ZO-1 protein, • reduction in Akt phosphorylation • production of pro-inflammatory cytokines TNF-α and IL-6 and NO	Propranolol improves postischemic hyperglycemia, impaired glucose tolerance and insulin resistance. Moreover, propranolol possesses anti-inflammatory, neuroprotective and hypoglycemic effects.
Manrique et al. (2011)	Nebivolol or placebo for 21 days in transgenic TG (mRen2)27 rat (Ren2)	Animal study	Nebivolol: • Improved insulin resistance, • decreased NADPH oxidase activity/levels.	Nebivolol has favorable effects on insulin resistance and oxidative stress.
Imbaby et al. (2014)	Group 1 - saline for 23 days parallel to DOX; Group 2 - DOX with normal saline daily; Groups 3 and 4 -curcumin for 30 days, starting 1 week before DOX; Groups 5 and 6 -nebivolol for 23 days, starting from the day of DOX administration; Group 7 - combination of curcumin and nebivolol in rats with doxorubicin-induced cardiac toxicity.	Animal study	Nebivolol alone or in combination with curcumin reduced redox biomarkers: • malondialdehyde, • glutathione peroxidase, • superoxide dismutase.	A combination of nebivolol and curcumin may be considered a potentially helpful candidate in the combination of chemotherapy with DOX to limit free radical-mediated organ injury.
Nascimento et al. (2022)	Control group - standard diet for 30 days; Group 2 - standard diet for 30 days added with nebivolol in the last 15 days; Group 3 - standard diet added with tenofovir for 30 days; Group 4 - standard diet added with tenofovir for 30 days and nebivolol in the last 15 days in rats with tenofovir-induced nephrotoxicity.	Animal study	Nebivolol: • restored thiobarbituric acid reactive substance levels, • reduced the renal protein expression (p47^phox^ and p67^phox^) and increased MnSOD and Nrf2.	Nebivolol attenuated tenofovir-induced nephrotoxicity and presented systemic and renal antioxidant, anti-inflammatory and anti-fibrotic properties.
Mollnau et al. (2003)	Nebivolol for 8 weeks in watanabe heritable hyperlipidemic rabbits	Animal study	Nebivolol: • inhibited phorbol ester-stimulated superoxide production, • improved NO downstream signaling (NOS uncoupling and normalization of NO/cGMP/cGK signaling)	Nebivolol may beneficially influence the progression of the atherosclerotic process.
Zhang et al. (2019)	Control group; DOX-treated group; DOX-treated mice with carvedilol; DOX-treated mice with carnosic acid and carvedilol/carnosic acid combination	Animal study	Carvedilol: • reduced serum levels of AST, ALT, lactate dehydrogenase and CK-MB, • increased levels of SOD, catalase, glutathion and NQO-1, • reduced levels of pro-inflammatory cytokines: COX2, TNF-α and IL-6 as well as NO.	Carvedilol and carnosic acid in combination could be expected to have synergistic efficacy and significant potential against cardiotoxicity induced by DOX.
Haas et al. (2016)	Carvedilol, propranolol and atenolol in the dextrose-induced endoplasmic reticulum (ER) stress in HepG2 cells	*In vitro*	Carvedilol, propranolol and atenolol: • inhibited endoplasmic reticulum stress, • superoxide production	The salutary effects of beta blockers on endothelial cells may contribute to the cardioprotective effects of these agents.
Zhang et al. (2022)	Carvedilol in retinal pigment epithelial cells induced with oxidative stress and apoptosis by high glucose	*In vitro*	Carvedilol: • reduced pro-inflammatory cytokines including TNF-α, IL-6 and IL-1β, • reduced redox biomarkers: SOD and glutathione peroxidase, • reduced apoptotic markers including Bax, cleaved-caspase 3, cleaved-caspase 9, • nhibited the inflammation, oxidative stress and apoptosis by activating the Nrf2/ARE signaling pathway.	Carvedilol effectively inhibited oxidative stress, apoptosis and cell damage in high glucose-induced retinal pigment epithelial cells, making it a promising future molecule for the treatment of diabetic retinopathy.

Akt, Protein kinase B; AST and ALT, aspartate and alanine aminotransferase; ARE, antioxidant responsive element; CD68, Cluster of Differentiation 68; CK-MB, creatine kinase isoenzyme-MB; COX-2, cyclooxygenase 2; CRP, C-reactive protein; DOX, doxorubicin; FRAP and GSSH, ratio, ferric-reducing ability of plasma and reduced glutathione/oxidized glutathione ratio; GFAP, glial fibrillary acidic protein; HDL, high-density lipoproteins; HOMA-IR, the homeostatic model assessment for insulin resistance; IL-1β, interleukin 1β; IL-6, interleukin 6; IRF8, interferon regulatory factor 8; LDL, low-density lipoproteins; MDA, malondialdehyde, mg/d–mg daily; SOD, superoxide dismutase; NOS, nitric oxide synthase; Nrf2, nuclear transcription factor of erythroid origin, type 2; NQO-1, NAD(P)H:quinone oxidoreductase-1; TNF-α, tumor necrosis factor-α; ZO-1, zonula occludens-1.

Rizos et al. conducted a pilot study comparing the metabolic profile of hypertensive patients with dyslipidemia when treated with nebivolol plus pravastatin versus atenolol plus pravastatin. Pravastatin is a member of the statin class of medications, primarily functioning as a competitive inhibitor of 3-hydroxy-3-methylglutaryl coenzyme A (HMG-CoA) reductase. This mechanism leads to a reduction in low-density lipoprotein (LDL) levels (Chen et al., 2017; Hague et al., 2016). Although pravastatin contribute to cardiovascular protection including improving of endothelial function, reducing inflammation, and present protective effects against cellular oxidative stress (Zhou and Liao, 2010; Higashi et al., 2010; Itani et al., 2020; Lu et al., 2015; Jang et al., 2020), on the other hand statins can influence glucose metabolism, potentially leading to increased blood glucose levels (Nayak, 2018). The study provided evidence that nebivolol, even in combination with pravastatin, has positive effect on glucose metabolism. Those findings render a nebivolol more appropriate therapy than atenolol for hypertensive patients suffering from metabolic syndrome or impaired glucose tolerance, potentially helping to stop the vicious cycle of hypertension, decreased insulin sensitivity, and hyperlipidemia (Rizos et al., 2003). A recent study conducted on a relatively small cohort (43 enrolled patients) indicated that nebivolol may improve insulin sensitivity and glucose utilization, potentially leading to better glycemic control in patients with diabetes mellitus or cardiometabolic syndrome. Additionally, nebivolol has been shown to improve insulin resistance related variables, fasting glucose, fasting insulin, and the homeostatic model assessment of insulin resistance (HOMA-IR) (Metwally et al., 2020). Marketou et al. conducted a systematic review and concluded that nebivolol has beneficial effects on insulin sensitivity and lipid metabolism due to its nitric oxide mediated properties (Marketou et al., 2017). Therefore, nebivolol may be a good therapeutic option for the treatment of hypertension in patients with disorders of glucose and lipid metabolism.

There is also evidence of a beneficial effect of “older” β-blockers on metabolic effects. Propranolol, a nonselective adrenergic receptor antagonist, has been shown to have a suppressive effect on hyperglycemia, inflammation and brain damage in a rat model of cerebral ischemia. Pre-treatment with propranolol protected against cerebral ischemia, edema and neuronal apoptosis. This neuroprotection was accompanied by reduced fasting glucose and insulin levels, impaired glucose tolerance, free fatty acids and corticosterone (Lin et al., 2020).

It is important to note that the influence of β-blockers on glucose metabolism and insulin sensitivity is complex and may vary based on factors such as the specific β-blocker used, patient population and underlying health conditions.

## 6 Oxidative stress and inflammation

Β-blockers have been studied for their potential impact on oxidative stress, a process characterized by an imbalance between the production of reactive oxygen species (ROS) and the body’s ability to detoxify these reactive intermediates (Table 1). Numerous factors have the capacity to provoke oxidative stress, including but not limited to diets rich in fats and sugars, exposure to radiation, thermal stress, specific chemical agents, tobacco use, and excessive intake of alcohol (Liu et al., 2024; Newsholme et al., 2016). Furthermore, within the context of cardiovascular disease (CVD), the aging of the cardiovascular system plays a crucial role as a contributing factor. On one hand, the aging process itself can lead to the development of oxidative stress due to structural and functional alterations that occur over time. Conversely, oxidative stress exacerbates this aging process by facilitating endothelial dysfunction, vascular remodeling, cardiac hypertrophy, and inflammation (Izzo et al., 2021).

Oxidative stress causes lipid peroxidation and ROS-mediated protein modifications, including the formation of cross-links and carbonyl groups or the breaking of polypeptide chains. Oxidative damage to DNA is mainly caused by the hydroxyl radical (OH). It primarily damages nitrogenous bases, which can then cause genetic mutations. Oxidative stress can not only damage cell components, but also disrupt cell differentiation/signaling pathways, or induce apoptosis. It is not surprising that oxidative stress has been implicated in a variety of pathological conditions, including cardiovascular diseases, diabetes and neurodegenerative disorders (Forman and Zhang, 2021; Maciejczyk et al., 2020).

### 6.1 β-blockers - potential mechanisms of oxidative stress and inflammation attenuation

**FIGURE 3:**
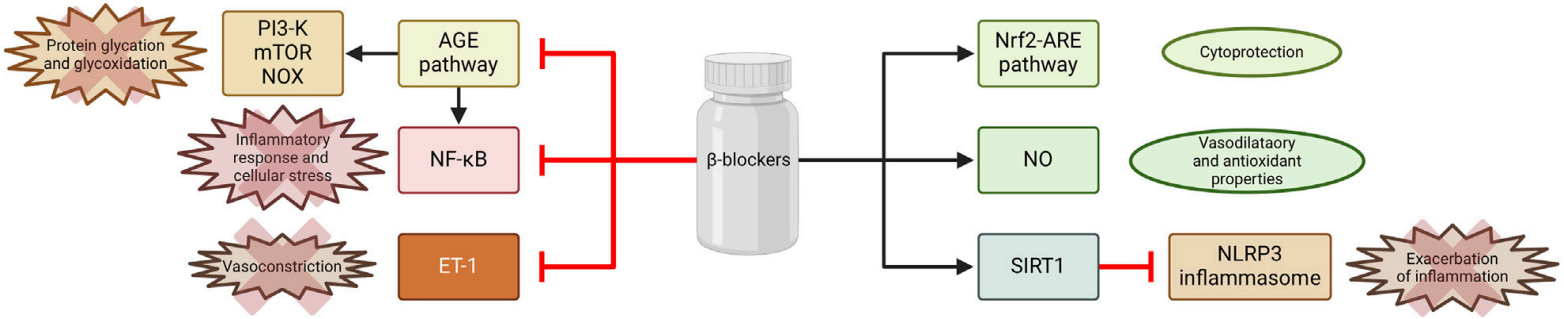
Selected mechanisms of inflammation attenuation by β -blockers. Created using biorender.com.

Activation of β1 adrenergic receptors by catecholamines stimulate nuclear factor kappa B (NF-κB) and the NLRP3 inflammasome within immune cells, particularly macrophages and neutrophils. This activation cascade produces pro-inflammatory cytokines, which are critical mediators of the inflammatory response. Furthermore, the activation of immune cells instigates the formation of reactive oxygen species (ROS), which can exacerbate tissue damage and inflammation. Concurrently, the stimulation of β2 adrenergic receptors contributes to the onset of endothelial dysfunction, characterized by impaired vascular integrity and increased permeability. This dysfunction is further associated with coagulopathy and thrombosis (Al-Kuraishy et al., 2021). By blocking β1 and β2 receptors, β-blockers may interact with NLRP3, potentially altering its activation or facilitating the activation of NLRP3 deactivators such as Sirtuin 1 (Wong et al., 2018). The Nrf2–ARE signaling pathway is a crucial defense mechanism against inflammation and oxidative stress. Nrf2 is a transcription factor that promotes the expression of a wide array of cytoprotective and detoxifying genes (Buendía et al., 2016). It was demonstrated that carvedilol can activate this pathway, thereby unveiling an additional mechanism through which inflammation and oxidative stress can be mitigated (Figure 3) (Zhang et al., 2022; Wang et al., 2014). One of the primary mechanisms by which β-blockers exert their anti-inflammatory effects involves the enhancement of nitric oxide (NO) release from endothelial cells. This effect is particularly pronounced with nebivolol, which activates endothelial nitric oxide synthase through β3-adrenergic receptor agonism, resulting in vasodilation and improved endothelial function. The increased availability of NO is essential, as it counteracts oxidative stress and inflammation, which are significant factors contributing to cardiovascular disease (Fongemie and Felix-Getzik, 2015; Howlett, 2014). Moreover, reducing vasoconstrictor tone represents another mechanism through which these beta-blockers enhance endothelial function. Nebivolol, carvedilol, and celiprolol have been shown to inhibit endothelin-1 (ET-1)-mediated vasoconstriction, a significant contributor to endothelial dysfunction in patients with hypertension (Saijonmaa et al., 1997; Tsubokou et al., 2002; Diehl et al., 2016). By decreasing ET-1 levels, β-blockers not only diminishes vasoconstriction but also augments the capacity for vasodilation, thereby improving endothelial responsiveness. Recent studies indicate that selected β-blockers may not only exhibit antioxidant activity, but also counteract protein glycation, which plays an important role in the pathogenesis of CVDs. Glycation refers to the non-enzymatic interaction between reducing sugars and amino acid residues in proteins, which leads to advanced glycation end-products (AGEs) (Yang et al., 2019). The interaction of AGEs with specific receptors, particularly the receptor for advanced glycation end-products (RAGE), triggers multiple intracellular signaling pathways, including NF-κB, ERK1/2, p38, and MAPK, which in turn promote inflammatory responses and cellular stress (Glynn et al., 2018; Yoshikawa et al., 2022; Yang et al., 2019). The AGE/RAGE signaling further amplifies the expression of cytokines and intensifies oxidative damage by activating NADPH oxidase (NOX) (Rezaeinezhad et al., 2021; Yang et al., 2019; Nesterowicz et al., 2023). Consequently, the processes of glycation and inflammation are closely interconnected, establishing a molecular framework for various diseases commonly associated with modern lifestyles. Lauko et al. showed in *in-vitro* study that concentrations of protein glycation products (Amadori products, β-amyloid, AGEs), glycooxidation products (dityrosine, kynurenine, N-formylkynurenine) and oxidation products (protein carbonyl groups, advanced oxidation protein products) decreased markedly in the presence of glycating agents (glucose, fructose, galactose, glyoxal and methylglyoxal) following the addition of propranolol (Lauko et al., 2024). The antiglycoxidative properties of the drug were similar to those of aminoguanidine, a known inhibitor of protein glycation and captopril, which is a recognized antioxidant. In silico analyses confirmed the antiglycation properties of propranolol during its interaction with glycosidases (e.g., α-amylase, α-glucosidase and sucrase-isomaltase) and proteins of the AGE/RAGE pathway, including RAGE, NF-kB, PI3-K and mTOR. It is suggested that the antiglycation action of propranolol may be due to its antioxidant properties. Propranolol showed strong antioxidant activity in ferric ion chelation and hydrogen peroxide scavenging assays, comparable to aminoguanidine and captopril.

### 6.2 Nebivolol

Nebivolol has been the subject of research regarding its potential effect on oxidative stress and its implications for various health conditions. Due to its NO-releasing capabilities, nebivolol has a nephroprotective action in hypertensive patients (Coats and Jain, 2017). In an animal model, nebivolol reduces doxorubicin (DOX) induced cardiotoxicity with a confirmed impact on redox biomarkers reduction (Imbaby et al., 2014). Furthermore, the unique vasodilating properties of this compound have been hypothesized to restore functional sympatholysis and improve muscle perfusion during exercise in hypertensive humans (Price et al., 2013). The positive effect of nebivolol on oxidative stress-related parameters was also observed in patients with uncomplicated mild-to-moderate essential hypertension (Zepeda et al., 2012). Nebivolol enhanced NO synthesis in tenofovir-induced nephrotoxicity and thus improved endothelial function, reduced superoxide infiltration in vessels and vascular macrophages, and prevented endothelial nitric oxide synthase (NOS III) uncoupling (Nascimento et al., 2022). Additionally, nebivolol inhibits NADPH oxidase activity in the blood and isolated neutrophils (Mollnau et al., 2003). It was also proven that other β-blockers (carvedilol, propranolol and atenolol) could potentially suppress dextrose-induced oxidative stress/endoplasmic reticulum stress and apoptosis in human coronary artery endothelial cells (Haas et al., 2016). Nebivolol may also modulate peroxynitrite induced inflammation and apoptosis, suggesting a role in reducing inflammation (Gandhi et al., 2008). The pleiotropic effects of nebivolol may not only result from the impact on intracellular signaling pathways. Of all the β-blockers, nebivolol has a symmetrical structure. It contains two benzopyran ring systems (Bekhradnia and Ebrahimzadeh, 2012), which is characteristic of compounds with antioxidant activity (Merken and Beecher, 2000; Koufaki et al., 2006).

### 6.3 Carvedilol

In the context of the impact of β-blockers on inflammation, most studies focus on carvedilol. The results of studies in both animal models and *in vitro* provide evidence that should be verified in well-designed clinical trials (Zhang et al., 2019; Fatani et al., 2015; El Morsy and Ahmed, 2020; Amirshahrokhi and Niapour, 2022; Savitz et al., 2000; Ronsein et al., 2005; Hassan et al., 2021). It has been proven that inflammatory response in DOX-induced cardiotoxicity was significantly suppressed by carvedilol combined with carnosic acid. Researchers observed reduced levels of pro-inflammatory cytokines (tumor necrosis factor-α (TNF-α), interleukin-6 (IL-6), interleukin-1β (IL-1β) and interleukin-18 (IL-18)), which were associated with inactivation of nuclear factor κB (NF-κB). Moreover, DOX-induced apoptosis and autophagy were dramatically attenuated thorough downregulation of cleaved caspase-3 and LC3B signaling pathways (Zhang et al., 2019). The effect of carvedilol on inflammation was also evaluated in experimentally induced ulcerative colitis in rats. The animals were pretreated with carvedilol for 7 days, and then ulcerative colitis was induced using acetic acid. The results showed that carvedilol attenuated lipid peroxidation and pro-inflammatory biomarkers and restored mucus content, sulfhydryl groups and antioxidant enzyme activity in the colon tissues. Furthermore, the protective effect of carvedilol was confirmed histologically, and was found to be similar to standard mesalazine therapy, suggesting its anti-inflammatory and antioxidant properties (Fatani et al., 2015). Another study with a similar approach investigated carvedilol’s role in L-arginine-induced acute pancreatitis. The research explored the mechanisms associated with carvedilol’s protection against L-arginine-induced acute pancreatitis and its impact on oxidative stress and inflammatory pathways. Carvedilol reduced the activity of α-amylase and lipase, as well as the levels of CRP and malondialdehyde. Carvedilol also significantly decreased NF-κB, mitogen-activated protein kinase p38, signal transducer and activator of transcription 1, TNF-α, IL-1β, myeloperoxidase (MPO) and phospholipase A2. Additionally, carvedilol reduced the expression of pancreatitis-associated protein 2 and platelet-activating factor genes. In summary, carvedilol protected against l-arginine-induced acute pancreatitis in rats by inhibiting cellular oxidative stress and inflammatory pathways, contributing to pancreatic damage (El Morsy and Ahmed, 2020). Some studies conducted in animal models suggested the protective effect of carvedilol in the brain of mice with hepatic encephalopathy. The compound induced the Nrf2/HO-1 pathway, which is involved in the defense against ROS overproduction. Not surprisingly, carvedilol reduced levels of oxidative stress markers in the brain of mice with hepatic encephalopathy. Additionally, carvedilol inhibited the activity of NF-κB and the expression of pro-inflammatory cytokines (TNF-α, IL1β and IL-6) in brain tissue, thereby reducing inflammation in hepatic encephalopathy. Furthermore, carvedilol led to a significant decrease in the levels of inflammatory mediators such as iNOS/NO, MPO, COX-2 and MCP-1 in the brain, indicating its anti-inflammatory effects in hepatic encephalopathy. Carvedilol also reduced cell death in the brain (increased the Bcl2/Bax ratio), demonstrating its neuroprotective properties (Amirshahrokhi and Niapour, 2022). These findings are consistent with previous research highlighting the neuroprotective effects of carvedilol, which demonstrated that carvedilol provides protection in focal cerebral ischemia, that is associated with reduced apoptosis and downregulation of inflammatory cytokines (Savitz et al., 2000). Furthermore, carvedilol has been shown to exert cytoprotective effects against ROS generation in the liver, indicating its antioxidant properties (Ronsein et al., 2005). Finally, Hassan et al.investigated the protective effects of carvedilol against hepatic ischemia-reperfusion injury in rats. The results demonstrated that hepatic ischemia-reperfusion led to increased oxidative stress, inflammatory responses and endothelial dysfunction. Carvedilol treatment mitigated hepatic enzyme damage, restored oxidative balance, modulated inflammatory state, and regulated endothelial (eNOS) and inducible (iNOS) nitric oxide synthase expression (Hassan et al., 2021).

## 7 Conclusion and future perspectives

Based on the review of the impact of β-blockers on glucose metabolism, insulin sensitivity, oxidative stress and inflammation, several conclusions and future perspectives can be drawn. As mentioned earlier, the influence of β-blockers on glucose metabolism, inflammation and oxidative stress is based on a complex mechanism and varies based on the specific β-blocker used, patient population and underlying health conditions. Researches have indicated that certain β-blockers, particularly nebivolol and carvedilol, may have favorable effects on metabolic parameters and glycemic control in patients with diabetes mellitus or cardiometabolic syndrome. Additionally, the vasodilatory and nitric oxide-mediated properties of nebivolol and carvedilol have been associated with potential benefits in improving insulin sensitivity and lipid metabolism in hypertensive patients with disorders of glucose and lipid metabolism. These findings suggest that vasodilating β-blockers may have potential therapeutic options for patients with metabolic disorders.

Moreover, β-blockers, especially those with vasodilatory properties, have been associated with antioxidant effects, potentially contributing to reducing oxidative stress markers. Nebivolol, in particular, has been the subject of multiple studies regarding its potential impact on oxidative stress, with several studies suggesting nephroprotective effects, reduction of myocardial apoptosis and improvement in endothelial function, all of which are related to its antioxidant properties. Carvedilol has also been shown to protect against oxidative stress and inflammation in various conditions, such as cardiotoxicity, ulcerative colitis, acute pancreatitis and hepatic ischemia-reperfusion injury. These findings highlight the potential role of β-blockers, especially nebivolol and carvedilol, in mitigating oxidative stress and inflammation implicated in various pathological conditions.

**FIGURE 4:**
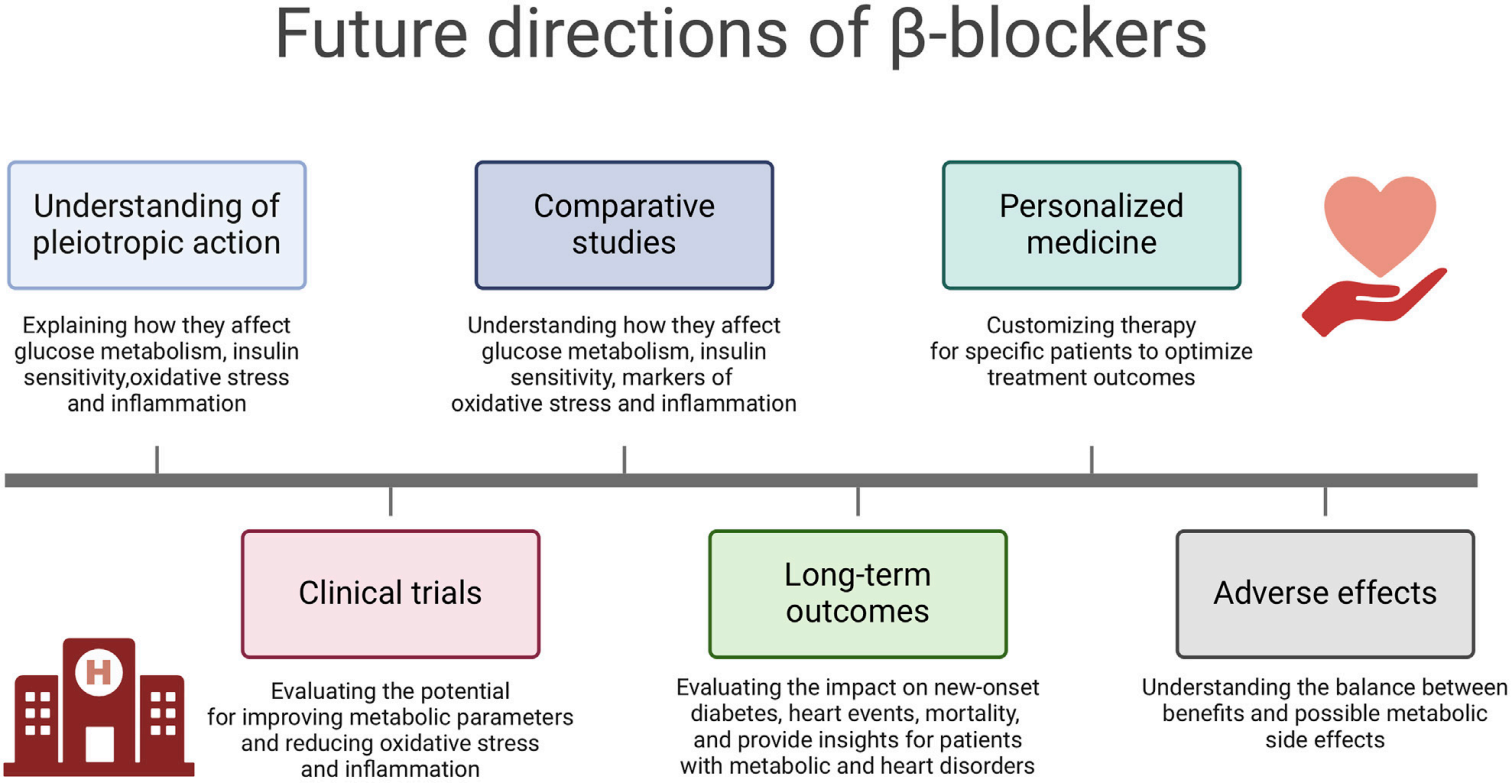
Future directions of β-blockers. Created using biorender.com.

It should be noted that many of the presented findings were based on animal model studies. In the case of studies conducted on humans or human biological material, the cohorts were represented by a limited number of patients. While for understanding disease mechanisms and testing therapies, the valuable animal models have limitations in their ability to fully replicate human conditions due to species differences. While these models can provide insights, they may only sometimes translate directly to human responses. Small cohort studies need to be more robust in generalizing findings due to limited samples, which can impact the sensitivity to detect differences, reducing the robustness of the findings. Based on above mentioned findings and presented limitations, several future perspectives can be considered (Figure 4):

1. Understanding of the pleiotropic action: Future research should focus on elucidating the specific molecular and cellular mechanisms through which β-blockers, particularly nebivolol and carvedilol, exert their effects on glucose metabolism, insulin sensitivity, oxidative stress and inflammation. Understanding these mechanisms at a fundamental level can provide insights into the development of targeted therapeutic strategies.
2. Clinical Trials: Well-designed clinical trials are warranted to further evaluate the therapeutic potential of β-blockers in improving metabolic parameters and reducing oxidative stress and inflammation in various disease states. These trials should consider different patient populations, including those with diabetes mellitus, metabolic syndrome and cardiovascular diseases.
3. Comparative Studies: Comparative studies evaluating the effects of different classes of β-blockers on glucose metabolism, insulin sensitivity, oxidative stress and inflammation markers can provide valuable information for clinical decision-making. Understanding the differential effects of β-blockers can help tailor treatment strategies to individual patient needs.
4. Long-term Outcomes: Long-term prospective studies are needed to assess the impact of β-blockers on the development of new-onset diabetes mellitus, cardiovascular events and overall mortality. These studies can provide valuable insights into the clinical implications of using β-blockers in patients with metabolic disorders and cardiovascular risk factors.
5. Personalized Medicine: Future research should explore the potential for personalized medicinal approaches using β-blockers based on individual patient characteristics, such as genetic factors, metabolic profiles and comorbidities. Tailoring β-blocker therapy to specific patient phenotypes may optimize treatment outcomes.
6. Adverse Effects: Further investigation into the potential adverse effects of β-blockers on glucose metabolism, insulin sensitivity and lipid metabolism is warranted. Understanding the balance between the beneficial effects and potential metabolic side effects of β-blockers is crucial for optimizing their clinical use.

The future perspectives outlined above can contribute to advancing our understanding of the role of β-blockers in modulating metabolic parameters and oxidative stress, ultimately guiding the development of more effective and personalized therapeutic strategies for patients with metabolic disorders and cardiovascular diseases.
